# Supplementary material for: Controlled edge dependent stacking of WS2-WS2 Homo- and WS2-WSe2 Hetero-structures: A Computational Study
Source: Sci Rep. 2020 Feb 3;10:1648. doi: 10.1038/s41598-020-58149-6 (PMC6997452; doi:10.1038/s41598-020-58149-6)
Supplement: Supplementary file 1 — Supplementary Information. [file 41598_2020_58149_MOESM1_ESM.docx]

**Supporting Information**

**Controlled edge dependent stacking of WS_2_-WS_2_ Homo- and WS_2_-WSe_2_ Hetero-structures: A Computational Study**

Kamalika Ghatak, Kyung Nam Kang, Eui-Hyeok Yang*, Dibakar Datta*

^1^ Department of Mechanical and Industrial Engineering, New Jersey Institute of Technology, Newark, NJ 07103, USA

^2^ Department of Mechanical Engineering, Stevens Institute of Technology, Hoboken, NJ 07030, USA

* Corresponding author

**Section S1. Scaling of the calculation setup (VASP):**

All our structures are non-periodic in nature, and therefore, it requires the increased simulation box size to avoid the error due to the periodic image overlap. We have utilized GPU facility of our institutional cluster KONG for VASP simulation. To scale the kinetic energy cut-off, we have considered one case with a total of 80 atoms (BL-*c*-AA-W_20_S_60_). For this case, a box size of (35× 35×35) Å with 4 combinations of energy cut-off starting from 300-600 eV (300, 400, 500, and 600 eV) were taken. We have performed 4 single point calculation, and the variation in energy values from 400 eV – 600 eV is insignificant. Therefore, we have considered 400 eV kinetic energy cut-off for all our computations. Moreover, this single point calculation (one geometric iteration) takes time in between ~2-3 hrs. and the time increases with the increasing kinetic cut-off values. These number of geometric iterations for all these structures vary in between ~200-400 geometric iterations depending upon the systems. There are few reasons behind this significant time requirement for all these geometries: a) the presence of heavy transition metal such as W, b) the huge box size, c) high kinetic energy cut-off and, finally d) the non-periodic nature of the calculation. Therefore, an optimized combination of all these issues need to be taken care of in order to resist the huge computational cost. In this regard, we first considered 2 combinations of the cubic cell sizes Comb-A (35 × 35×35) Å^3^ and, Comb-B (27× 27×27) Å^3^ and ran a single point calculation to trace the time consumption (row 1 and 2 of Table 1). In these two cases, the average wall time/ iteration is huge, and it will take a lot of time in order to get the full optimization. Therefore, after several trials and error, we have optimized the simulation box size to be Comb-C (24× 22×20) Å^3^ (row 3 of Table S1), where the distance of the two consecutive periodic images were kept more than 8 Å. The initial lengths of the triangular *c*-terminated and *m*-terminated flakes are ~13.2 Å, and ~11.8 Å.

**Table S1.** Cell size parametrization for TMD BL optimization.


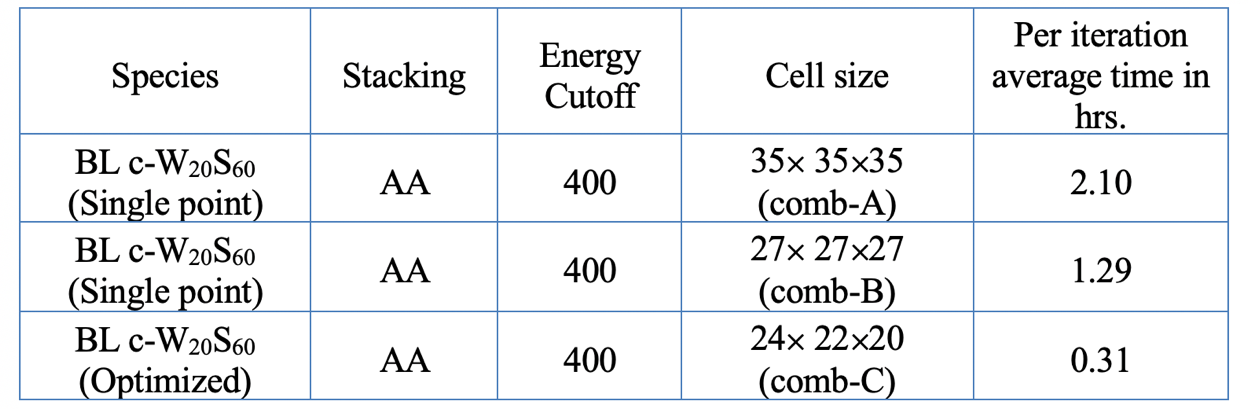


**Section S2. Labelled optimized geometries of the ML & BL TMDs:**


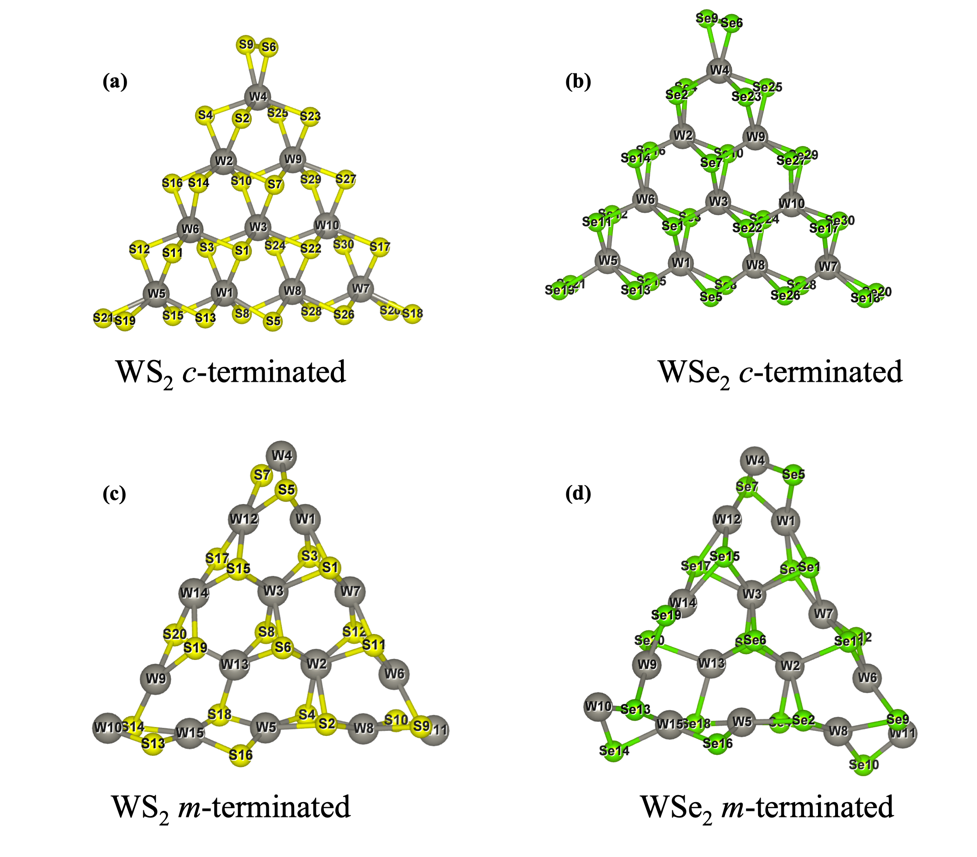


**Fig. S1.** Optimized labelled top views of a) *c*- terminated WS_2_, b) *c*-terminated WSe_2_, c) *m*-terminated WS_2_ and, d) *m*-terminated WSe_2_.


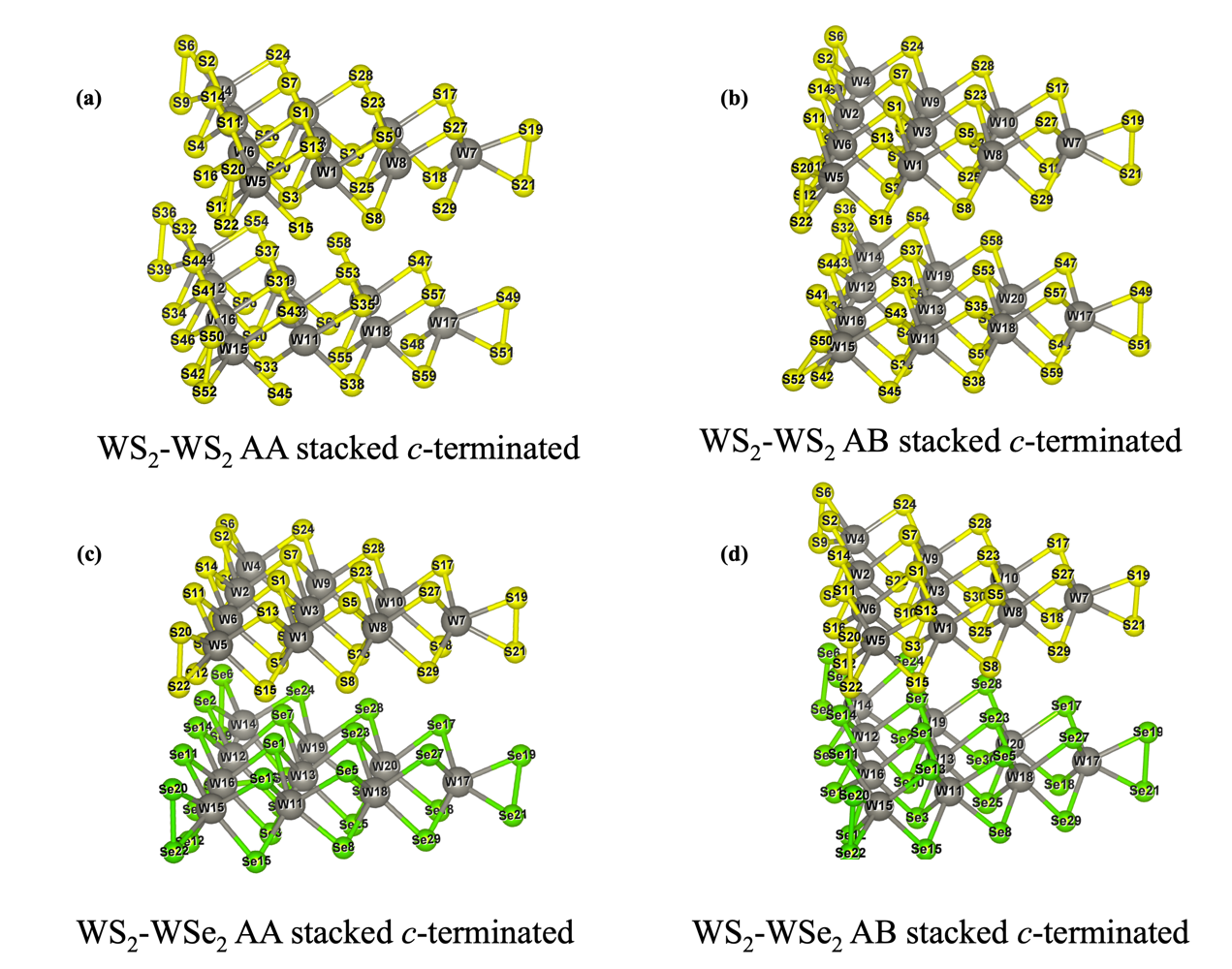


**Fig. S2.** Optimized labelled side views of a) *c*- terminated AA stacked WS_2_-WS_2_, b) *c*- terminated AB stacked WS_2_-WS_2_, c) *c*- terminated AA stacked WS_2_-WSe_2_ and, d) *c*- terminated AB stacked WS_2_-WSe_2_.


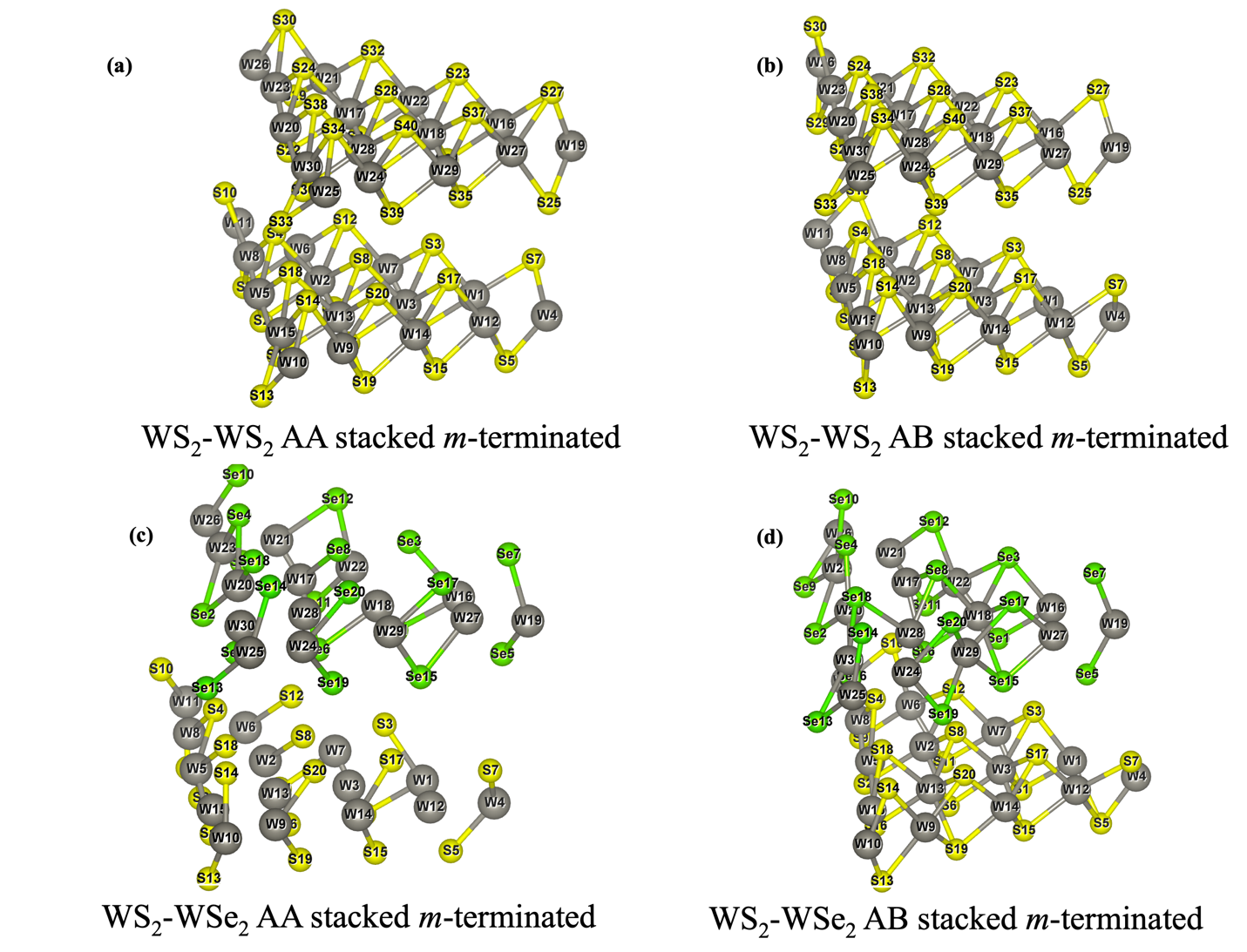


Fig. S3. Optimized labelled side views of a) *m*- terminated AA stacked WS_2_-WS_2_, b) *m*- terminated AB stacked WS_2_-WS_2_, c) *m*- terminated AA stacked WS_2_-WSe_2_ and, d) *m*- terminated AB stacked WS_2_-WSe_2_.

**Section S3. Bader Charge Analysis for the corresponding systems:**

All the charges were tabulated based on the atoms number provided on Fig S2 and S3. The highest and lowest positively charged atom’s electron partition were given in **Red** color and the negatively charged highest and lowest atom’s electron partition were provided in **Green** color. Their corresponding charges were provided inside the bracket and in **Blue** color.

| **WS2-WS2 AA stacked c-terminated** |  |
| --- | --- |
| *Atom number* | *Bader charge* |
| W_1 | 3.7922 |
| W_2 | 3.7589 |
| W_3 | 3.9923 |
| W_4 | 3.4615 |
| W_5 | 3.5019 |
| W_6 | 3.758 |
| W_7 | 3.5093 |
| W_8 | 3.8072 |
| W_9 | 3.7804 |
| W_10 | 3.7658 |
| W_11 | 3.7746 |
| W_12 | 3.8198 |
| W_13 | **4.0273 (+1.9727 e)** |
| W_14 | 3.4712 |
| W_15 | 3.5118 |
| W_16 | 3.804 |
| W_17 | **3.2494 (+2.7506 e)** |
| W_18 | 3.7994 |
| W_19 | 3.7856 |
| W_20 | 3.7683 |
| S_1 | 6.9879 |
| S_2 | 6.8927 |
| S_3 | 7.0206 |
| S_4 | 6.8634 |
| S_5 | 6.8692 |
| S_6 | 6.3651 |
| S_7 | 6.9586 |
| S_8 | 6.7752 |
| S_9 | 6.3498 |
| S_10 | 7.0261 |
| S_11 | 6.8733 |
| S_12 | 6.8514 |
| S_13 | 6.8569 |
| S_14 | 6.839 |
| S_15 | 6.7738 |
| S_16 | 6.728 |
| S_17 | 6.8421 |
| S_18 | 6.8119 |
| S_19 | **6.2582 (-0.2582 e)** |
| S_20 | 6.2695 |
| S_21 | 6.4046 |
| S_22 | 6.4253 |
| S_23 | 6.9701 |
| S_24 | 6.8541 |
| S_25 | 7.0187 |
| S_26 | 6.7725 |
| S_27 | 6.8647 |
| S_28 | 6.8109 |
| S_29 | 6.7748 |
| S_30 | 6.7844 |
| S_31 | 7.0063 |
| S_32 | 6.8741 |
| S_33 | 6.9732 |
| S_34 | 6.8698 |
| S_35 | 6.7764 |
| S_36 | 6.3423 |
| S_37 | 7.0006 |
| S_38 | 6.8332 |
| S_39 | 6.3697 |
| S_40 | 6.9707 |
| S_41 | 6.8096 |
| S_42 | 6.8324 |
| S_43 | 6.7561 |
| S_44 | 6.7076 |
| S_45 | 6.8631 |
| S_46 | 6.8219 |
| S_47 | 6.829 |
| S_48 | **7.1884 (-1.884 e)** |
| S_49 | 6.2587 |
| S_50 | 6.3462 |
| S_51 | 6.399 |
| S_52 | 6.3202 |
| S_53 | 7.012 |
| S_54 | 6.7907 |
| S_55 | 6.9717 |
| S_56 | 6.8171 |
| S_57 | 6.7525 |
| S_58 | 6.778 |
| S_59 | 6.8661 |
| S_60 | 6.8319 |
| WS2-WS2 AB stacked c-terminated |  |
| Atom number | Bader electron Charge partition |
| W_1 | 3.776 |
| W_2 | 3.7835 |
| W_3 | 4.028 (+1.972 e) |
| W_4 | 3.1061 (+2.8939 e) |
| W_5 | 3.3837 |
| W_6 | 3.7844 |
| W_7 | 3.1572 |
| W_8 | 3.861 |
| W_9 | 3.7764 |
| W_10 | 3.7824 |
| W_11 | 3.7439 |
| W_12 | 3.8124 |
| W_13 | 4.011 |
| W_14 | 3.1863 |
| W_15 | 3.271 |
| W_16 | 3.7953 |
| W_17 | 3.2411 |
| W_18 | 3.8144 |
| W_19 | 3.8133 |
| W_20 | 3.7385 |
| S_1 | 6.9723 |
| S_2 | 7.1269 (-1.1269 e) |
| S_3 | 6.9892 |
| S_4 | 6.7431 |
| S_5 | 6.7817 |
| S_6 | 6.2849 |
| S_7 | 6.9731 |
| S_8 | 6.7843 |
| S_9 | 6.3191 |
| S_10 | 6.99 |
| S_11 | 6.7422 |
| S_12 | 6.8866 |
| S_13 | 6.8061 |
| S_14 | 6.851 |
| S_15 | 6.9563 |
| S_16 | 6.8279 |
| S_17 | 7.0715 |
| S_18 | 6.7637 |
| S_19 | 6.262 (-0.262 e) |
| S_20 | 6.429 |
| S_21 | 6.4072 |
| S_22 | 6.2779 |
| S_23 | 6.9811 |
| S_24 | 7.1218 |
| S_25 | 6.98 |
| S_26 | 6.7545 |
| S_27 | 7.0399 |
| S_28 | 6.826 |
| S_29 | 6.7648 |
| S_30 | 6.8089 |
| S_31 | 7.0074 |
| S_32 | 6.7348 |
| S_33 | 6.9775 |
| S_34 | 7.0797 |
| S_35 | 6.751 |
| S_36 | 6.3303 |
| S_37 | 7.0178 |
| S_38 | 6.7889 |
| S_39 | 6.3761 |
| S_40 | 6.9771 |
| S_41 | 7.1069 |
| S_42 | 6.7671 |
| S_43 | 6.8052 |
| S_44 | 6.8094 |
| S_45 | 6.8459 |
| S_46 | 6.8256 |
| S_47 | 6.9622 |
| S_48 | 6.7488 |
| S_49 | 6.3112 |
| S_50 | 6.4066 |
| S_51 | 6.3402 |
| S_52 | 6.392 |
| S_53 | 6.9823 |
| S_54 | 6.7491 |
| S_55 | 6.9809 |
| S_56 | 6.972 |
| S_57 | 7.1025 |
| S_58 | 6.8249 |
| S_59 | 6.7743 |
| S_60 | 6.8637 |
| WS2-WSe2 AA stacked c-terminated |  |
| Atom_number | Bader electron Charge partition |
| W_1 | 3.797 |
| W_2 | 3.7474 |
| W_3 | 3.9959 |
| W_4 | 3.5276 (+2.4724 e) |
| W_5 | 3.3683 |
| W_6 | 3.8111 |
| W_7 | 3.5447 |
| W_8 | 3.7824 |
| W_9 | 3.7726 |
| W_10 | 3.7833 |
| W_11 | 4.3316 |
| W_12 | 4.3383 |
| W_13 | 4.5441 (+1.4559) |
| W_14 | 3.9669 |
| W_15 | 4.1049 |
| W_16 | 4.3398 |
| W_17 | 4.0082 |
| W_18 | 4.3071 |
| W_19 | 4.3402 |
| W_20 | 4.3256 |
| S_1 | 6.9896 |
| S_2 | 6.9105 |
| S_3 | 7.0642 |
| S_4 | 6.874 |
| S_5 | 6.9015 |
| S_6 | 6.3639 |
| S_7 | 6.977 |
| S_8 | 6.8133 |
| S_9 | 6.364 |
| S_10 | 7.0421 |
| S_11 | 6.9375 |
| S_12 | 6.8194 |
| S_13 | 6.9777 |
| S_14 | 6.8338 |
| S_15 | 6.8115 |
| S_16 | 6.7619 |
| S_17 | 6.851 |
| S_18 | 6.8159 |
| S_19 | 6.4178 |
| S_20 | 6.4134 |
| S_21 | 6.2778 |
| S_22 | 6.3209 |
| S_23 | 6.9926 |
| S_24 | 6.8791 |
| S_25 | 7.0672 (-1.0672 e) |
| S_26 | 6.8285 |
| S_27 | 6.8574 |
| S_28 | 6.8456 |
| S_29 | 6.871 |
| S_30 | 6.7766 |
| Se_1 | 6.7351 |
| Se_2 | 6.669 |
| Se_3 | 6.7035 |
| Se_4 | 6.5131 |
| Se_5 | 6.5011 |
| Se_6 | 6.3058 |
| Se_7 | 6.7287 |
| Se_8 | 6.594 |
| Se_9 | 6.284 |
| Se_10 | 6.719 |
| Se_11 | 6.5896 |
| Se_12 | 6.5702 |
| Se_13 | 6.622 |
| Se_14 | 6.525 |
| Se_15 | 6.5722 |
| Se_16 | 6.6337 |
| Se_17 | 6.524 |
| Se_18 | 6.6461 |
| Se_19 | 6.3137 |
| Se_20 | 6.2616 |
| Se_21 | 6.3028 |
| Se_22 | 6.2462 (-0.2462 e) |
| Se_23 | 6.7441 |
| Se_24 | 6.6438 |
| Se_25 | 6.7278 |
| Se_26 | 6.5215 |
| Se_27 | 6.6308 |
| Se_28 | 6.534 |
| Se_29 | 6.5993 |
| Se_30 | 6.6446 |
| WS2-WSe2 AB stacked c-terminated |  |
| Atom_number | Bader electron Charge partition |
| W_1 | 3.8172 |
| W_2 | 3.8055 |
| W_3 | 4.0469 |
| W_4 | 3.0693 |
| W_5 | 3.0431 (+2.9569 e) |
| W_6 | 3.7794 |
| W_7 | 3.5063 |
| W_8 | 3.7834 |
| W_9 | 3.7883 |
| W_10 | 3.7839 |
| W_11 | 4.3183 |
| W_12 | 4.3459 |
| W_13 | 4.5561(+1.4439 e) |
| W_14 | 4.0523 |
| W_15 | 4.074 |
| W_16 | 4.3104 |
| W_17 | 4.0825 |
| W_18 | 4.3383 |
| W_19 | 4.3219 |
| W_20 | 4.2984 |
| S_1 | 6.9737 |
| S_2 | 7.1226 |
| S_3 | 7.0423 |
| S_4 | 6.7926 |
| S_5 | 6.83 |
| S_6 | 6.333 |
| S_7 | 6.9874 |
| S_8 | 6.8127 |
| S_9 | 6.3199 |
| S_10 | 7.0374 |
| S_11 | 7.0652 |
| S_12 | 6.7993 |
| S_13 | 7.1308 |
| S_14 | 6.8391 |
| S_15 | 6.813 |
| S_16 | 6.7888 |
| S_17 | 6.8474 |
| S_18 | 6.8381 |
| S_19 | 6.3101 |
| S_20 | 6.3536 |
| S_21 | 6.4066 |
| S_22 | 6.42 |
| S_23 | 6.9631 |
| S_24 | 7.1364 (-1.1364 e) |
| S_25 | 7.0396 |
| S_26 | 6.796 |
| S_27 | 6.849 |
| S_28 | 6.8576 |
| S_29 | 6.9085 |
| S_30 | 6.8442 |
| Se_1 | 6.7079 |
| Se_2 | 6.4865 |
| Se_3 | 6.7408 |
| Se_4 | 6.6889 |
| Se_5 | 6.554 |
| Se_6 | 6.2752 |
| Se_7 | 6.7092 |
| Se_8 | 6.6152 |
| Se_9 | 6.2383 |
| Se_10 | 6.7337 |
| Se_11 | 6.6231 |
| Se_12 | 6.5508 |
| Se_13 | 6.6329 |
| Se_14 | 6.5276 |
| Se_15 | 6.5986 |
| Se_16 | 6.6331 |
| Se_17 | 6.5621 |
| Se_18 | 6.56 |
| Se_19 | 6.2596 |
| Se_20 | 6.2189 (-0.2189 e) |
| Se_21 | 6.2993 |
| Se_22 | 6.321 |
| Se_23 | 6.7176 |
| Se_24 | 6.5026 |
| Se_25 | 6.7311 |
| Se_26 | 6.6858 |
| Se_27 | 6.6488 |
| Se_28 | 6.542 |
| Se_29 | 6.6215 |
| Se_30 | 6.6344 |

| **WS_2_-WS_2_ AA stacked *m*-terminated** |  |
| --- | --- |
| *Atom_number* | *Bader Charge* |
| W_1 | 4.5663 |
| W_2 | 4.2158 |
| W_3 | 4.2431 |
| W_4 | 4.7545 |
| W_5 | 4.3288 |
| W_6 | 4.6824 |
| W_7 | 4.4091 |
| W_8 | 4.628 |
| W_9 | 4.5508 |
| W_10 | 4.8743 |
| W_11 | 4.8599 |
| W_12 | 4.6719 |
| W_13 | 4.14 |
| W_14 | 4.4481 |
| W_15 | 4.6043 |
| W_16 | **3.3687 (+2.6313 e)** |
| W_17 | 4.2024 |
| W_18 | 4.1992 |
| W_19 | 4.8176 |
| W_20 | 4.3493 |
| W_21 | 4.5339 |
| W_22 | 4.4124 |
| W_23 | 4.7856 |
| W_24 | 4.6933 |
| W_25 | 4.9682 |
| W_26 | 4.8853 |
| W_27 | **5.8745 (+0.1255 e)** |
| W_28 | 4.1641 |
| W_29 | 4.303 |
| W_30 | 4.622 |
| S_1 | 7.0966 |
| S_2 | 7.1012 |
| S_3 | 7.1106 |
| S_4 | 7.1054 |
| S_5 | 7.1241 |
| S_6 | **6.9867 (-0.9867 e)** |
| S_7 | **7.2023 (-1.2023 e)** |
| S_8 | 7.0216 |
| S_9 | 7.1046 |
| S_10 | 7.1554 |
| S_11 | 7.0629 |
| S_12 | 7.1326 |
| S_13 | 7.1085 |
| S_14 | 7.0579 |
| S_15 | 7.0783 |
| S_16 | 7.0714 |
| S_17 | 7.0967 |
| S_18 | 7.1049 |
| S_19 | 7.1292 |
| S_20 | 7.1387 |
| S_21 | 7.1225 |
| S_22 | 7.1246 |
| S_23 | 7.0965 |
| S_24 | 7.0561 |
| S_25 | 7.1245 |
| S_26 | 7.009 |
| S_27 | 7.0983 |
| S_28 | 7.011 |
| S_29 | 7.1537 |
| S_30 | 7.0837 |
| S_31 | 7.1102 |
| S_32 | 7.0801 |
| S_33 | 7.1494 |
| S_34 | 7.0445 |
| S_35 | 7.1335 |
| S_36 | 7.0984 |
| S_37 | 7.0793 |
| S_38 | 7.0923 |
| S_39 | 7.1077 |
| S_40 | 7.0784 |
| WS2-WS2 AB stacked m-terminated |  |
| Atom_number | Bader Charge |
| W_1 | 4.4886 |
| W_2 | 4.2469 |
| W_3 | 4.2146 |
| W_4 | 4.8674 |
| W_5 | 4.4671 |
| W_6 | 4.6867 |
| W_7 | 4.2574 |
| W_8 | 4.5256 |
| W_9 | 4.4837 |
| W_10 | 4.869 |
| W_11 | 4.9025 |
| W_12 | 4.7257 |
| W_13 | 4.2139 |
| W_14 | 4.3839 |
| W_15 | 4.6559 |
| W_16 | 5.8867 (+0.1133 e) |
| W_17 | 4.1813 |
| W_18 | 4.1681 |
| W_19 | 4.8505 |
| W_20 | 4.3921 |
| W_21 | 4.5962 |
| W_22 | 4.3804 |
| W_23 | 4.6607 |
| W_24 | 4.7543 |
| W_25 | 4.896 |
| W_26 | 4.8297 |
| W_27 | 3.3663 (+2.6337 e) |
| W_28 | 4.1753 |
| W_29 | 4.4569 |
| W_30 | 4.5708 |
| S_1 | 7.1052 |
| S_2 | 7.1353 |
| S_3 | 7.1525 |
| S_4 | 7.1465 (-1.1465 e) |
| S_5 | 7.083 |
| S_6 | 6.9985 |
| S_7 | 7.1324 |
| S_8 | 7.019 |
| S_9 | 7.0602 |
| S_10 | 7.0799 |
| S_11 | 7.0661 |
| S_12 | 7.1753 |
| S_13 | 7.0803 |
| S_14 | 7.0791 |
| S_15 | 7.0987 |
| S_16 | 7.112 |
| S_17 | 7.0955 |
| S_18 | 7.1075 |
| S_19 | 7.1464 |
| S_20 | 7.138 |
| S_21 | 7.0561 |
| S_22 | 7.0859 |
| S_23 | 7.1043 |
| S_24 | 7.0741 |
| S_25 | 7.0517 |
| S_26 | 6.9975 (-0.9975 e) |
| S_27 | 7.1033 |
| S_28 | 7.0087 |
| S_29 | 7.1075 |
| S_30 | 7.1329 |
| S_31 | 7.1196 |
| S_32 | 7.1283 |
| S_33 | 7.1278 |
| S_34 | 7.072 |
| S_35 | 7.1308 |
| S_36 | 7.0992 |
| S_37 | 7.1386 |
| S_38 | 7.1046 |
| S_39 | 7.1049 |
| S_40 | 7.0866 |
| WS2-WSe2 AA stacked m-terminated |  |
| Atom_number | Bader Charge |
| W_1 | 4.6471 |
| W_2 | 4.2029 (+1.7971 e) |
| W_3 | 4.2598 |
| W_4 | 4.8404 |
| W_5 | 4.308 |
| W_6 | 4.6683 |
| W_7 | 4.4788 |
| W_8 | 4.6546 |
| W_9 | 4.3201 |
| W_10 | 4.9066 |
| W_11 | 4.7937 |
| W_12 | 4.5668 |
| W_13 | 4.1933 |
| W_14 | 4.329 |
| W_15 | 4.773 |
| W_16 | 4.9187 |
| W_17 | 4.8499 |
| W_18 | 4.762 |
| W_19 | 5.1762 (+0.8238 e) |
| W_20 | 4.8153 |
| W_21 | 4.7564 |
| W_22 | 4.7219 |
| W_23 | 4.9417 |
| W_24 | 4.871 |
| W_25 | 5.1712 |
| W_26 | 5.1122 |
| W_27 | 4.8267 |
| W_28 | 4.6797 |
| W_29 | 4.7626 |
| W_30 | 4.8875 |
| S_1 | 7.1153 |
| S_2 | 7.0836 |
| S_3 | 7.1358 |
| S_4 | 7.091 |
| S_5 | 7.0858 |
| S_6 | 7.0045 |
| S_7 | 7.1355 |
| S_8 | 7.0303 |
| S_9 | 7.0921 |
| S_10 | 7.1645 |
| S_11 | 7.0719 |
| S_12 | 7.1066 |
| S_13 | 7.0974 |
| S_14 | 7.0696 |
| S_15 | 7.1399 |
| S_16 | 7.0856 |
| S_17 | 7.1278 |
| S_18 | 7.0921 |
| S_19 | 7.1499 |
| S_20 | 7.1658 (-1.1658 e) |
| Se_1 | 6.8588 |
| Se_2 | 6.8967 |
| Se_3 | 6.8725 |
| Se_4 | 6.8416 |
| Se_5 | 6.8907 |
| Se_6 | 6.7453 |
| Se_7 | 6.7924 |
| Se_8 | 6.756 |
| Se_9 | 6.8906 |
| Se_10 | 6.7651 |
| Se_11 | 6.814 (-0.814 e) |
| Se_12 | 6.8606 |
| Se_13 | 6.8941 |
| Se_14 | 6.8145 |
| Se_15 | 6.8262 |
| Se_16 | 6.8417 |
| Se_17 | 6.8302 |
| Se_18 | 6.8661 |
| Se_19 | 6.8744 |
| Se_20 | 6.8278 |
| WS2-WSe2 AB stacked m-terminated |  |
| Atom_number | Bader Charge |
| W_1 | 4.7064 |
| W_2 | 4.2549 |
| W_3 | 4.1701 |
| W_4 | 4.8452 |
| W_5 | 4.5263 |
| W_6 | 4.6113 |
| W_7 | 4.3729 |
| W_8 | 4.7298 |
| W_9 | 5.9104 (+0.0896 e) |
| W_10 | 4.848 |
| W_11 | 4.7699 |
| W_12 | 4.6159 |
| W_13 | 4.2307 |
| W_14 | 4.3277 |
| W_15 | 3.2404 (+2.7596 e) |
| W_16 | 4.8426 |
| W_17 | 4.8483 |
| W_18 | 4.6918 |
| W_19 | 5.1919 |
| W_20 | 4.5752 |
| W_21 | 4.9508 |
| W_22 | 4.81 |
| W_23 | 4.9561 |
| W_24 | 4.7622 |
| W_25 | 5.1297 |
| W_26 | 4.9477 |
| W_27 | 4.7798 |
| W_28 | 4.8141 |
| W_29 | 4.8387 |
| W_30 | 4.8014 |
| S_1 | 7.0792 |
| S_2 | 7.0448 |
| S_3 | 7.1231 |
| S_4 | 7.0686 |
| S_5 | 7.0683 |
| S_6 | 6.9849 |
| S_7 | 7.1724 (-1.1724 e) |
| S_8 | 7.0104 |
| S_9 | 7.1215 |
| S_10 | 7.1466 |
| S_11 | 7.0623 |
| S_12 | 7.1017 |
| S_13 | 7.0826 |
| S_14 | 7.0867 |
| S_15 | 7.0803 |
| S_16 | 7.1168 |
| S_17 | 7.1104 |
| S_18 | 7.1305 |
| S_19 | 7.0833 |
| S_20 | 7.1158 |
| Se_1 | 6.8252 |
| Se_2 | 6.9682 |
| Se_3 | 6.9057 |
| Se_4 | 6.8218 |
| Se_5 | 6.8117 |
| Se_6 | 6.6598 |
| Se_7 | 6.8225 |
| Se_8 | 6.7499 (-0.7499 e) |
| Se_9 | 6.9719 |
| Se_10 | 6.8909 |
| Se_11 | 6.8703 |
| Se_12 | 6.851 |
| Se_13 | 6.9173 |
| Se_14 | 6.8237 |
| Se_15 | 6.8648 |
| Se_16 | 6.872 |
| Se_17 | 6.8819 |
| Se_18 | 6.8758 |
| Se_19 | 6.8956 |
| Se_20 | 6.8299 |
